# Medical infrared thermal imaging of syringomyelia in the Cavalier King Charles Spaniel

**DOI:** 10.1186/s12917-020-02354-y

**Published:** 2020-05-14

**Authors:** M. Larkin, C. Loughin, D. Marino, C. Dewey, S. Umbaugh, J. Sackman

**Affiliations:** 1 Department of Surgery, Long Island Veterinary Specialists, 163 South Service Road, Plainview, NY 11803 USA; 2 The Canine Chiari Institute at Long Island Veterinary Specialists, 163 South Service Road, Plainview, NY 11803 USA; 3 Department of Clinical Sciences, Cornell University College of Veterinary Medicine, 930 Campus Road, Box 33, Ithaca, NY 14853 USA; 4 Computer Vision and Image Processing Laboratory, Electrical and Computer Engineering Department, Southern Illinois University Edwardsville, Edwardsville, IL 62062 USA

**Keywords:** Medical infrared thermal imaging, Thermography, Chiari-like malformation, Syringomyelia, Syrinx

## Abstract

**Background:**

Medical infrared thermal imaging (MITI) is a non-invasive imaging modality gaining popularity in the veterinary field. An infrared camera captures emission of heat and creates a color map in the form of a thermogram. Topical heat emission is influenced by localized disease processes as a result of autonomic nervous system imbalance. The purpose of this study was to determine the utility of using thermography to identify changes in thermographic patterns associated with syringomyelia (SM) presence or absence in Cavalier King Charles Spaniels (CKCS) with Chiari-like Malformation (CLM).

**Results:**

In CKCS with CLM, MITI was most accurate at a texture distance of 6. Optimizing imaging feature sets produced a highest accuracy of 69.9% (95% CI: 59.5–79.0%), with 81.3% sensitivity and 57.8% specificity for identifying the presence of syringomyelia.

**Conclusion:**

Thermographic image analysis is a successful non-invasive, diagnostic test that can be used to screen for syringomyelia presence in a CKCS with CLM.

## Background

Chiari-like malformation (CLM) is a neurologic condition affecting the craniocervical junction in dogs. Increasing evidence suggests that CLM in dogs is a condition in which the entire skull is malformed, although the caudal aspect is the most obvious abnormality on MRI [1]. If the abnormality is severe enough, there can be cerebellar herniation through the foramen magnum [2]. Syringomyelia (SM) is often found in association with CLM. SM is characterized by one or more fluid-containing cavities (syrinx) within the parenchyma of the spinal cord [3]. Syrinx formation secondary to CLM is not fully understood but is believed to be multifactorial including structural abnormalities coupled with changes in CSF flow, blood flow and pressures [4]. The majority of patients with CLM only have symptoms after the development of SM [5]. Dogs affected with CLM/SM can experience debilitating neuropathic discomfort and neurologic abnormalities.

The CLM/SM malformation is found most commonly in the Cavalier King Charles Spaniel (CKCS). It has been determined to be heritable and as many as 85% of CKCS are affected [6–8]. The most common clinical sign noted is pain secondary to disruption of the fibers of the dorsal horn laminae [9–11]. Other clinical sings include; scratching (also termed “air-guitar” or “phantom scratching”) [11], allodynia (pain from non-noxious stimuli), hyperesthesia sensitivity to touch, facial rubbing, ataxia, paresis, exercise intolerance and lethargy [2, 3, 5].

Diagnosis of CLM/SM is supported by history, signalment, clinical signs, and diagnostic imaging findings. Imaging modalities for dogs experiencing cervical pain include: contrast myelography, radiography, computed tomography (CT), and magnetic resonance imaging (MRI). MRI is considered the modality of choice in humans & veterinary medicine when investigating a clinical suspicion of CLM or SM. MRI has significant limitations in veterinary medicine including: expense, requirement for general anesthesia, and limited availability.

Medical infrared thermal imaging (MITI), also known as medical infrared imaging (MII) or infrared thermography (IRT), is a noninvasive imaging modality used for screening purposes in human medicine [12] and gaining popularity in veterinary medicine [13]. MITI requires use of a specific camera that captures emission of heat from the surface of objects, such as skin. The sympathetic portion of the autonomic nervous system directly controls local dermal microcirculation and is responsible for the surface heat detected [14, 15]. Once the thermal radiation is captured, a thermal pattern is then generated in the form of a color map called a thermogram. Warm regions (typically colored white, red, or orange) are associated with increased circulation or metabolic rates secondary to disease processes (clinically associated with inflammation). Cold regions (typically colored blue or black) are associated with reduced perfusion suggestive of infarction or autonomic system imbalance. Therefore, thermography can be used to quantitatively detect alterations in sympathetic tone (i.e., surface heat distribution) that typically occurs secondary to injury or other disease processes [16]. MITI has been used successfully in small animal veterinary medicine to evaluate numerous conditions such as intervertebral disc disease [15], canine elbow dysplasia [17], feline hyperthyroidism [18] and cranial cruciate disease [19]. MITI has also been shown to be a useful method for estimating or even detecting cases of syringomyelia in human medicine [20]. MITI has many advantages over MRI including the lack of requirement for general anesthesia or sedation, less expensive equipment, rapid image acquisition time and decreased client expense.

The purpose of this retrospective study was to determine the utility of using MITI to identify differences in thermographic patterns associated with presence or absence of syringomyelia in CKCS affected with CLM. We hypothesized that the presence of SM secondary to CLM will be associated with a recognizable change in thermographic pattern.

## Results

### Demographics

One hundred dogs were included in this study. Due to poor image quality (usually motion artifact) preventing proper interpretation on some of the MITI images, seven of the dogs were eliminated totaling 93 CKCS in this study. Of the 93 CKCS included, the average age was 33.7 months old (SD = 27.4 months) and the average weight of each patent was 8.1 kg (17.8 pounds). There were 35 female intact (37.6%), 24 female spayed (25.8%), 15 male intact (16.1%) and 19 male neutered (20.4%) CKCS. Of the 93 CKCS included in this study, all (100%) had CLM based on complete, full-body MR imaging; 48 (51.6%) were diagnosed with SM and 45 (48.4%) had no evidence of SM.

### Image pattern analysis

**Fig. 1:**
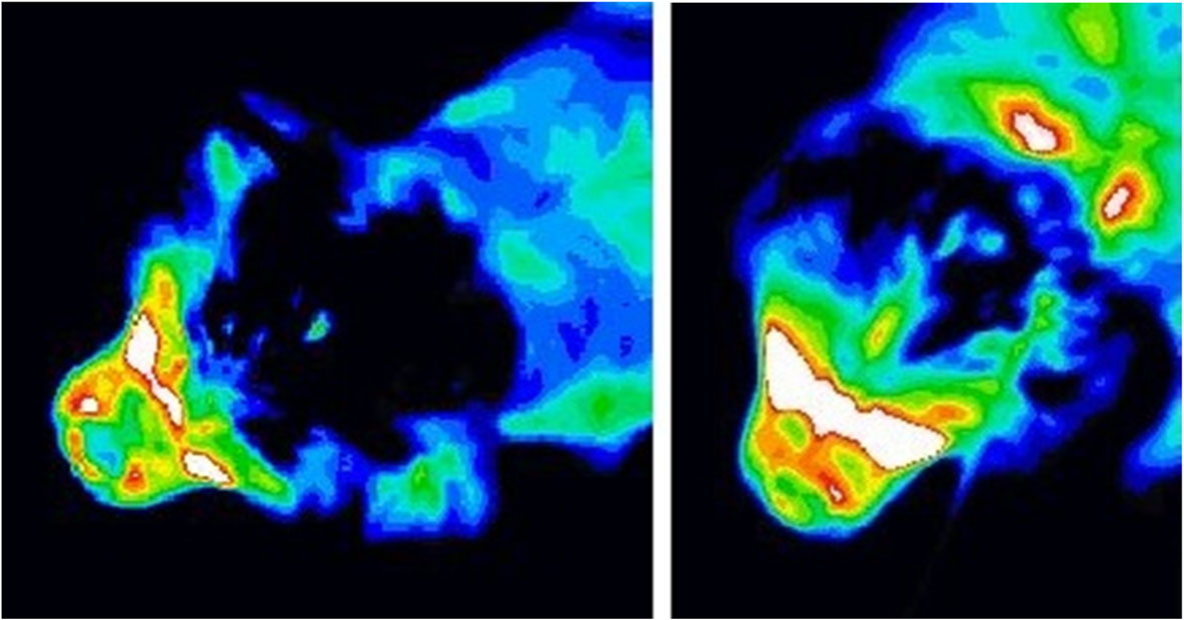
Thermogram of a CKCS (dorsal head view). The image on the left represents a CKCS without syringomyelia. The image on the right represents a CKCS with syringomyelia

There were 8184 experiments of various parameters performed. Calculated statistics, average and standard deviation for all 8184 permutations were performed. The top ten best results for each texture distance (6, 7, 8 and 9) were documented. During the experiment, different feature sets yielded different results as expected. The most effective features for syrinx identification were found to be histogram mean, histogram energy, texture inertia, texture energy, texture correlation, and texture entropy. Similarly, the experiments show Euclidean distance to be the best comparison metric and K-nearest neighbor as the optimal classification method. Standard normal density and softmax scaling normalization methods were both shown to be useful. Figure 1 shows thermograms for two different CKCS without (left) and with (right) syringomyelia.

The best results were achieved using a texture distance of 6 (Table 1). The highest accuracy rate among feature sets at this texture distance was approximately 70% (65 out of the 93 images correctly identified as SM present or absent; 95% CI: 59.5–79.0%). Depending on features used, pattern recognition analysis achieved a sensitivity as high as 83.3% (correctly identifying 40 of the 48 images that had syrinx presence; 95% CI: 69.8–92.5%) in one trial, and also achieved a specificity as high as 73.3% (correctly identifying 33 of the 45 images with syrinx absence; 95% CI: 58.1–85.4%) in another trial.

**Table 1:** Thermographic image pattern recognition: the top ten best results from a texture distance of six

Feature Set*	# correct (out of 93)	% correct (out of 93) - accuracy	Syrinx presence - # correct (out of 48)	Syrinx presence - % sensitivity	Syrinx absence - # correct (out of 45)	Syrinx absence - % specificity
A	65	69.89%	39	81.25%	26	57.78%
B	64	68.82%	40	83.33%	24	53.33%
C	64	68.82%	33	68.75%	31	68.89%
D	64	68.82%	32	66.67%	32	71.11%
E	64	68.82%	31	64.58%	33	73.33%
F	63	67.74%	39	81.25%	24	53.33%
G	63	67.74%	36	75.00%	27	60.00%
H	63	67.74%	35	72.92%	28	62.22%
I	63	67.74%	34	70.83%	29	64.44%
J	63	67.74%	33	68.75%	30	66.67%

Feature sets included one or more of the following features: histogram mean, histogram energy, texture inertia, texture energy, texture correlation, and texture entropy

## Discussion

MITI using pattern recognition software was successfully used to differentiate between thermographic patterns of CKCS dogs with CLM and SM vs. those without SM. We found MITI using pattern recognition software was 70% accurate in correctly identifying CKCS dogs with CLM and SM with an 81% sensitivity and 58% specificity. As such, these results reaffirm the view of MITI as a screening tool rather than a diagnostic tool given the high sensitivity and relatively low specificity. Additional studies are being conducted with different mask sizes (region of interest, as defined in methods section) and histogram features to see if better improvements in sensitivity and specificity can be made. By reducing the mask size (i.e., eliminating muzzles, ears, etc.), the background data or “noise” (artifact) can be reduced thus concentrating the more relevant data and potentially allowing for improved feature identification. Larger data sets including normal are being acquired, as this information will enhance the accuracy of the normal masks making comparisons more distinguishable.

MITI may be helpful clinically for clients who decline advanced diagnostic imaging due to financial concerns, patient anesthetic risk concerns or other reasons. MITI might provide further insight for these non-surgical candidates and aid in appropriate, goal-directed therapy.

The diagnosis of CLM can only be made by MRI, which is also the preferred imaging modality for diagnosing SM. There has been some acceptance in the veterinary community of the use of “abbreviated” MRI studies utilizing views of the head and neck region with implementation of limited imaging sequences in an effort to reduce costs. SM has been reported to be most commonly located at the C1–4 and T2-L2 spinal cord segments in a prior study [21]. MITI could be used as a screening tool to further support the necessity of full spinal imaging.

Historically, a goal of MITI has been to identify changes associated with pathology prior to clinical signs [22–24]. With further improvements in sensitivity, MITI has potential to be offered as a low-cost screening tool for breeders in young patients. Young patients can be imaged easily for the development of syringomyelia in patients without clinical signs, with continued monitoring as growth occurs. MITI might aid in determining CKCS breed reproduction standards and sales of healthy offspring in the future. Further investigation with regards to MITI in young CKCS dogs is needed.

Treatment options for CLM/SM involve surgical or medical management. The preferred method of treating humans with CLM/SM is surgical decompression via a procedure called foramen magnum decompression (FMD) [25–27]. The authors have reported surgical success in veterinary patients as well, with a FMD combined cranioplasty procedure that was able to improve or resolve clinical signs in approximately 80% of dogs [28, 29]. Further studies are on going to evaluate for syrinx resolution in these post-operative patients. Currently, the only method of reassessing syrinx status after surgery is to repeat a full spinal MRI. MITI could potentially be used as a screening for syringomyelia resolution in post-operative FMD patients. Further development of computer recognition pattern analysis to improve both sensitivity and specificity would be necessary.

This study revealed that MITI is a successful screening test for the presence of SM in CKCS with CLM. Compared to other imaging modalities, MITI is a quick, inexpensive modality that does not require sedation nor anesthesia, and eliminates radiation exposure (to patient & staff). MITI does not provide insight to syrinx location or severity nor should it be used as a sole diagnostic modality. While veterinary thermographic imaging continues to improve as advances in technology occur, MRI will remain the gold standard for definitive diagnosis and staging of Chiari-like malformation and syringomyelia.

### Limitations

Limitations of this study include the moderate sample/population size, limited number of thermographic views obtained, and only one disease process (CLM) presence.

In this study, dorsal craniocervical views were chosen based on a pilot study utilizing four views (front of head, top of head, left side of head, right side of head) that revealed that the top of the head view provided the best success rate (unpublished author data). Secondly, the top of the head view includes the craniocervical junction most appropriately compared to other views. Imaging of this region was important, as it has been shown that CSF obstruction at the foramen magnum causes turbulent flow that is associated with SM presence and severity [30]. It was, and still is, believed that this turbulent flow at the foramen magnum accounts for changes noted in our thermographic patterns seen with syringomyelia presence. Ongoing studies are currently investigating spinal thermographic imaging (dorsal full spinal views) in an attempt to improve overall success rate for SM identification in the CKCS.

All included dogs were diagnosed with CLM via MRI and excluded if other abnormalities or disease processes were detected. Given this study design, it is not known whether it is possible for MITI to distinguish SM from other cervical pathologies without MRI confirmation. In future studies, it would be useful to image patients with other pathologies as well as absence of pathology for control purposes. Despite all dogs having an MRI confirmed diagnosis of CLM, these dogs were not further divided into different categories based on their CLM disease. Divided categories could include CLM compression severity (mild, moderate, severe), or craniocervical compression types (medullary kinking, dorsal compression, etc.) [31].

Future studies based on larger populations, data sets (including normal and other pathologies), and mask sizes may help further improve pattern recognition software success.

It is worth noting that although MITI is not readily available in all veterinary clinics, its growing popularity has made obtaining the necessary equipment more feasible for most practices. Compared to other diagnostic imaging modalities, MITI requires significantly less equipment and minimal space occupancy. Computer software packages for image processing are readily available to all.

## Conclusion

We concluded that MITI was successful in detecting thermographic pattern changes associated with syringomyelia in CKCS affected with CLM. MITI should serve as a screening test for the presence or absence of SM, and would work best in conjunction with MRI. Future studies should focus on improving overall success rate, identifying syrinx location and quantifying syrinx severity. Future studies using MITI to determine its viability for tracking SM disease progression and/or resolution could also be beneficial. Improvements in sensitivity and specificity for SM identification are desirable.

## Methods

### Case selection

The medical records of 216 dogs diagnosed with CLM from 2007 to 2010 at The Canine Chiari Institute at Long Island Veterinary Specialists (Plainview, NY) were reviewed. One hundred CKCS dogs that were diagnosed with CLM were included in this study.

All dogs had a complete physical & neurologic evaluation performed by a Diplomate of the American College of Veterinary Surgeons. All dogs had full brain/spinal MRI, a craniocervical CT scan, cranial cervical radiography, and craniocervical thermal imaging during that time. These dogs were also found to have no comorbidities (systemically or discovered on imaging) and also had no prior surgical interventions for any disease process. After imaging and recovery from anesthesia all dogs were discharged to the care of their respective owners.

### Thermographic imaging

All dogs had restricted exercise and were allowed to acclimate to the room temperature (21 °C) for 30 min prior to MITI. Images were obtained using an infrared camera (Med 2000 IRIS, Meditherm, Inc. Beaufort, NC) with a focal plane array amorphous silicone microbolometer. Images were obtained from the top of the head (dorsal view), approximately one meter away from the patient. All dogs were imaged unsedated in sternal recumbency. Color coding was selected so that warm regions appear white, red or orange and cold regions appear blue or black. To minimize thermal artifact from handling, trained technicians wearing latex gloves positioned dogs without touching the head and cervical regions. A uniform board was placed under each patient to minimize background artifact. To further aid in minimizing artifact, users would custom select specific regions of interest on the acquired thermogram. These outlined regions of interest are known as “masks”. Masks provide the opportunity to exclude accidentally incorporated extremities, etc., and focus on the relevant portion of an image. In this study, masks were created to include the entire head and cervical region only. All images were sent to the Department of Electrical and Computer Engineering, School of Engineering, Southern Illinois University, Edwardsville, IL for evaluation. After thermographic imaging, all dogs were then placed under general anesthesia for radiographic, MRI, and CT imaging.

### MRI imaging

Magnetic resonance imaging of the brain and spinal cord of each dog were acquired by use of an Achieva 3.0-T MR imaging unit (Philips, Andover, MA). Imaging was performed in dorsal recumbency with the neck in partial flexion (between 100^°^ and 138°) to approximate the craniocervical angle in standing CKCS as previously described [31]. Sagittal T2-weighted, transverse T2-weighted, and transverse T1-weighted fluid-attenuated inversion recovery images of the brain were acquired in each dog. In addition, sagittal T2-weighted and transverse T2-weighted images of the entire cervical, thoracic and lumbar regions were acquired in each dog. Presence or absence of syringomyelia was confirmed with MRI.

### CT imaging

Computed tomographic images of the head and cranial vertebral column including C3 were acquired by use of a multidetector CT (Marconi Mx8000, Marconi Medical Systems Inc., Cleveland, OH) operating at 140 kV and 150 mA; a bone reconstruction filter was used during image acquisition. Dogs were placed in sternal recumbency with the neck in partial flexion (between 100° and 138°). Continuous 1 mm collimated images were obtained by use of helical acquisition.

### Thermographic software

Images were evaluated at the Computer Vision and Image Processing Laboratory at Southern Illinois University. A software program (WINTES2, Compix Inc., Lake Oswego, OR) was used to save, analyze and review image data. The different color maps were converted and remapped to 256-level gray scale images by linearly mapping the temperature range of 19 °C to 40 °C, as this temperature range covers the entire range of the body temperature of canines. From there, histograms are then automatically created. A histogram is a plot of the gray level values versus the number of pixels at that value [32]. Custom processing software (CVIPtools) was used for image pattern recognition and to analyze and evaluate thermographic patterns.

### Imaging pattern analysis

Utilizing the image processing software described above, thermographic images were independently analyzed with computer recognition pattern analysis (CRPA) to differentiate between the presence or absence of syringomyelia. The primary tools used include Analysis Features and Analysis Pattern Classification. Data normalization methods included soft-max with R = 1 and standard normal density normalization. The CRPA assesses and sorts different image features found between the two groups (presence or absence of SM). Image features that were assessed included histogram features and texture features. Histogram features are statistical-based features, where the histogram is used as a model of the probability distribution of the gray levels (providing information about the characteristics of the image) [32]. Histogram features include mean (general brightness of an image), standard deviation (image contrast), skew (asymmetry about the mean), energy (gray level distribution) and entropy (image complexity) [32]. Texture features are related to properties such as smoothness, coarseness, roughness and regular patterns [32]. The five most useful texture features have been found to be energy (measures homogeneity or smoothness), inertia (measure of contrast), correlation (measures similarity between pixels), inverse difference (measure for local homogeneity), and entropy (measures information content) [32]. Texture distance is the pixel distance between pairs of pixels. Texture distances of 6, 7, 8 and 9 were used in this study. Texture distance chosen depends on the resolution of the image and the coarseness of the texture of interest [32]. Two classification methods (K-Nearest Neighbor with K = 3 and Nearest Neighbor) were also assessed. Evaluation for optimal image, feature and classification method were based on accuracy, sensitivity, and specificity compared to MRI results, as calculated with the leave-one-out cross-validation method. Confidence intervals for proportions were calculated based on the binomial probability distribution.

## Data Availability

The datasets used and/or analyzed during the current study are available from the corresponding author upon reasonable request.

## Abbreviations

CKCS: Cavalier King Charles Spaniels
CLM: Chiari-like malformation
CRPA: Computer recognition pattern analysis
CSF: Cerebral spinal fluid
CT: Computed tomography
FMD: Foramen magnum decompression
MITI: Medical infrared thermal imaging
MRI: Magnetic resonance imaging
SM: Syringomyelia
